# Mapping of HNF4α target genes in intestinal epithelial cells

**DOI:** 10.1186/1471-230X-9-68

**Published:** 2009-09-17

**Authors:** Mette Boyd, Simon Bressendorff, Jette Møller, Jørgen Olsen, Jesper T Troelsen

**Affiliations:** 1 Department of Cellular and Molecular Medicine. Panum Institute, Building 6.4. University of Copenhagen. Blegdamsvej 3B 2200 Copenhagen N, Denmark

## Abstract

**Background:**

The role of HNF4α has been extensively studied in hepatocytes and pancreatic β-cells, and HNF4α is also regarded as a key regulator of intestinal epithelial cell differentiation. The aim of the present work is to identify novel HNF4α target genes in the human intestinal epithelial cells in order to elucidate the role of HNF4α in the intestinal differentiation progress.

**Methods:**

We have performed a ChIP-chip analysis of the human intestinal cell line Caco-2 in order to make a genome-wide identification of HNF4α binding to promoter regions. The HNF4α ChIP-chip data was matched with gene expression and histone H3 acetylation status of the promoters in order to identify HNF4α binding to actively transcribed genes with an open chromatin structure.

**Results:**

1,541 genes were identified as potential HNF4α targets, many of which have not previously been described as being regulated by HNF4α. The 1,541 genes contributed significantly to gene ontology (GO) pathways categorized by lipid and amino acid transport and metabolism. An analysis of the homeodomain transcription factor Cdx-2 (*CDX2*), the disaccharidase trehalase (*TREH*), and the tight junction protein cingulin (*CGN*) promoters verified that these genes are bound by HNF4α in Caco2 cells. For the Cdx-2 and trehalase promoters the HNF4α binding was verified in mouse small intestine epithelium.

**Conclusion:**

The HNF4α regulation of the Cdx-2 promoter unravels a transcription factor network also including HNF1α, all of which are transcription factors involved in intestinal development and gene expression.

## Background

The intestinal epithelium continuously renews its cells by division of a stem/progenitor cell population located in the crypts. The daughter cells rapidly expand by cell divisions and migrate from the crypt to villus. The cells finally differentiate into the mature cell types of the intestine. In the small intestine these cells are enterocytes, paneth cells, goblet cells, and enteroendocrine cells. In the colon two major cell types predominate: colonocytes and goblet cells. The differentiation state of the intestinal cells can be determined by their location on the crypt/villus axis. Cells located in the bottom of the crypts are undifferentiated and proliferate (except for the paneth cells, which are located in the very bottom of the crypt). The cells located in the upper crypt and on the villus are differentiated and express digestive enzymes, transport proteins, mucins, or hormones, depending on the cell type.

The differentiation process of the intestinal epithelium is highly organised and regulated at the transcriptional level [1]. A few transcription factors regulating the differentiation-dependent transcription have been described. Cdx-2 is a homeodomain transcription factor, which in the adult mouse is only expressed in the intestine [2], and has been reported to regulate the expression of several intestinal specific genes, like lactase-phlorizin hydrolase (*LCT*)[3], sucrase-isomaltase (*SI*) [4], calbindin D9k (*S100G*) [5,6], hephaestin (*HEPH*) [7], IL-Cadherin (*CDH17*) [8], and phospholipase (*PLA2G12B*) [9]. Inactivation of the Cdx-2 gene results in an inability of the epithelial cells to differentiate [10], and overexpression can force the undifferentiated intestinal cell line IEC-6 [11] to differentiate. HNF1α has also been found to regulate several intestinal-specific genes often in combination with Cdx-2 [9,12-18], but inactivation of the HNF1α gene in transgenic mice only causes minor changes in the intestinal transcription.) [19]. GATA-factors seem to be important regulators of the longitudinal expression pattern of some genes [13,15,18,20-25]. HNF4α is another transcription factor expressed in the intestine. HNF4α has been shown to be important for hepatic epithelium development [26]. Conditional inactivation of HNF4α gene in the colon in mice resulted in a failure to develop crypts, and a series of intestinal expressed genes were affected by the lack of HNF4α expression [27].

We have suggested that HNF4α is a main player in the transcriptional regulation of the small intestinal differentiation-dependent expression in mice, as promoters for genes that are up-regulated during the enterocyte differentiation have an over-representation of HNF4α sites in their promoters [28,29]. In the intestinal epithelium HNF4α is expressed along the entire length of the crypt villus axis except in the very bottom of the crypt, and it is therefore unlikely that HNF4α alone is responsible for the spatial restriction of gene expression to villus enterocytes [28,29]. Furthermore, it has been shown that HNF4α promotes differentiation of intestinal epithelial cells in a coculture system [30]. The HNF4α activity has been reported to be regulated on several different levels. CREB-binding protein (CBP) possesses an intrinsic acetyltransferase activity capable of acetylating HNF4α on lysine residues within the nuclear localization sequence. CBP-mediated acetylation increases the nuclear retention and the DNA binding activity of HNF4α, which increases the target gene activation by HNF4α [31]. It has also been reported that the expression or the activity of HNF4α can be modulated by signaling kinase activities like PKA [32], MAP kinase [33], and AMP-activated protein kinase [34]. Furthermore, several different isoforms are produced from the HNF4α gene by different promoter usage and 3'-end splicing codes [35]. Very little is known about the developmental and physiological relevance of the HNF4α isoforms.

To address the role of HNF4α for the differentiation-dependent transcription in human epithelial cells, we performed a genome-wide identification of promoters that are occupied by HNF4α *in vivo *in Caco-2 cells by the chromatin immunoprepitation followed by a DNA promoter array analysis (ChIP-chip analysis). The ChIP-chip analysis revealed that HNF4α is likely to be associated with the promoter regions of more than a thousand genes, which are mainly involved in transport and metabolism. New and interesting transcription factor networks were revealed by the analysis, most notably that HNF4α is found to regulate the expression of Cdx-2, and a model is proposed in which HNF4α initiates the differentiation-dependent transcription by simultaneously activating HNF1α and Cdx-2, transcription factors necessary for the expression of many genes specifically expressed in the intestine [36].

## Methods

### Plasmid construction

All primers used in this study were purchased from MWG Biotech. The primers in additional file 1 were used to amplify the following promoter sequences from Human Genomic DNA (Roche): *CDX2 *(GenBank no. NM_001265) position -1212 to +36. *CGN *(GenBank no. NM_020770) position -1343 to +25. *TREH *(GenBank no. NM_007180) position -558 to +84. For each promoter the PCR product was TA cloned in pCR^®^2.1 (Invitrogen). The cloned PCR fragment was cloned into pGL4.10 (Promega) using the *XhoI *and *HindIII *or *BglII *sites introduced by the primers. Site-directed mutagenesis was done with all three plasmids to generate the corresponding promoter plasmids containing a mutated HNF4α binding site. The PCR product was cloned into the original plasmid replacing the wild-type sequence using the *XhoI *and *HindIII *or *BglII *sites. The primers used are listed in additional file 1. All the constructed plasmids were sequenced and analyzed on an ABI 3100 sequencer (Applied Biosystems).

### Cell cultures and transfections

Caco-2 and COS7 cells were cultured as monolayer on normal cell culture dishes in Dulbecco minimal essential medium containing 10% fetal calf serum and 100 μg/ml each of penicillin and streptomycin in a humidified atmosphere containing 5% CO. The media was changed twice a week. Caco-2 and COS7 cells were plated one day before transfection in 24-well plates (5 × 10^4 ^cells/well). The cells were transfected using 5 μL 5.47 mmol/L linear polyethylenimine (Exgen500; Fermentas) and 0.3 μg plasmid per well. A total of 50 ng of luciferase reporter plasmid and 12.5 ng of β-galactosidase expression plasmid (pCMVlacZ, Promega) were used in each transfection. The DNA concentrations were adjusted to 0.3 μg with pBluescript SK+ (Stratagene). The cells were harvested and analyzed two days after transfection for luciferase and β-galactosidase activity using the Dual Light system (Applied Biosystems). The luciferase measurements were normalized and corrected for transfection efficiencies using the β-galactosidase activities.

### RNA extraction and GeneChip hybridization

Total RNA was harvested from undifferentiated preconfluent Caco-2 cells and from differentiated Caco-2 cells (10 days after confluence) using the RNeasy kit (Qiagen). Biotin labelled cRNA was prepared as previously described [28] and hybridized to the Human Genome U133 Plus 2.0 Array (Affymetrix). Three independent GenecChip hybridisations were performed with RNA from both undifferentiated and differentiated Caco-2 cells. GeneChip data were analyzed by the robust multiarray analysis (RMA) procedure [37] using the implementation of RMA provided by the open source bioconductor project http://www.bioconductor.org[38]. The gene expression data is available at NCBI's GEO http://www.ncbi.nlm.nih.gov/sites/entrez?db=gds under the series accession number: GSE7745.

### Western blot

Western blot analyses were performed by separating nuclear extracts prepared as described in [39] from undifferentiated pre-confluent and differentiated 10-days post-confluent Caco-2 cells on a NuPAGE 12% Bis-Tris gel (Invitrogen). After electrophoresis, the gel was electrotransfered onto Immobilon membrane (Millipore). Immunoblotting of HNF4α was performed using 30 μg protein of Caco-2 nuclear extracts and a 100-fold dilution of HNF4α antibody sc-8987 (Santa Cruz). Immunoblotting of glyceraldehyde-3-phosphate dehydrogenase (*GAPD*) was performed using 10 μg protein of Caco-2 nuclear extracts and 1000× fold dilution of *GAPD *antibody(Chemicon). The blot was developed using the ECL kit (Amersham Biosciences) and chemiluminescence signals were captured using a LAS-1000+ (Fujifilm).

### Chromatin immunoprecipitation (ChIP) assay

#### Crosslinking and sonication

Caco-2 cells were cultured as described above until 10 days after confluence. At that stage the Caco-2 cells are expressing markers of the differentiated enterocyte, e.g. lactase phlorizin hydrolase [40]. Formaldehyde (Merck) was added to the culture medium to a final concentration of 1%. Cross-linking was allowed to proceed for 30 minutes at room temperature and stopped by addition of glycine to a final concentration of 0.125 M. This was followed by an additional incubation for five minutes. Fixed cells were rinsed twice with cold PBS (137 mM NaCl, 2.7 mM KCl, 43 mM Na_2_HPO_4_·7H_2_O, 1.4 mM KH_2_PO_4 _and 1 μl/ml protease inhibitors (Sigma Aldrich)) and harvested in cold PBS containing protease inhibitors. Cells were pelleted by centrifugation and resuspended in 6 ml SDS buffer (1% SDS, 10 mM EDTA, 50 mM Tris-HCl pH 8.0, and protease inhibitors). Cells were disrupted by sonication with a Branson Digital Sonifier 450, at 50% output for 15 seconds, followed by 30 seconds' resting time. This was repeated six times, yielding genomic DNA fragments with an average size of 500 bp.

Adult female mice were sacrificed by cervical dislocation. The small intestine was rapidly dissected. Small intestinal mucosa was scraped off with a microscope slide and frozen in liquid nitrogen. The frozen mucosa was pulverized with a mortar and added into 6 ml PBS containing 1% formaldehyde. The fixation was stopped after 30 min. by adding glycine to a final concentration of 0.125 M. The fixed mucosa was pelleted and sonicated as described for the Caco2 cells.

#### Immunoprecipitation

The immunoprecipitations and array promoter array analyses were done in triplicates. For each immunoprecipitation, 300 μL of crosslinked sonicated sample was diluted with 1.2 ml ChIP buffer (1% triton X-100, 150 mM NaCl, 2 mM EDTA, 20 mM Tris-HCl pH 8.1 and protease inhibitors (Sigma Aldrich)) and precleared for one hour by adding 50 μL of protein A beads (50% slurry protein A-Sepharose, Amersham; 0.5 mg/mL lipid-free BSA, (Sigma Aldrich); and 0.2 mg/mL salmon sperm DNA in TE). From each sample 15 μl, corresponding to 1% of the input DNA, was removed for later use as input control. Samples were immunoprecipitated overnight at 4°C with polyclonal antibodies specific for either HNF-4α (4 μg, Santa Cruz, Cat. no. sc-8987), HA (4 μg, Santa Cruz, Cat. no. sc-805) or acetylated Histone H3 (10 μg, Upstate Biotech, Cat. no. 06-599). Immune complexes from the Caco-2 chromatin were recovered by adding 50 μL of protein A beads (50% slurry protein A-Sepharose, Amersham; 0.5 mg/mL lipid-free BSA, 0.2 mg/mL salmon sperm DNA in TE) and incubated for two hours at 4°C. Immune complexes from mouse small intestine were isolated using 50 μL of protein A magnetic beads (Dynal, Invitrogen). The beads were washed twice in Wash Buffer 1 (0.1% SDS, 1% Triton X-100, 0.1 Deoxycholate, 150 mM NaCl, 1 mM EGTA, 2 mM EDTA, 20 mM Tris-HCl pH 8.0), twice in Wash Buffer 2 (0.1% SDS, 1% Triton X-100, 0.1% Deoxycholate, 500 mM NaCl, 1 mM EGTA, 2 mM EDTA, 20 mM Tris-HCl pH 8.0), once in Wash Buffer 3 (0.25 M LiCl, 0.5% Deoxycholate, 0.5% NP-40, 0.5 mM EGTA, 1 mM EDTA, 10 mM Tris-HCl pH 8.0), and twice in TE buffer. The DNA was eluted by adding 300 μL elution buffer (1% SDS and 0.1 M NaHCO_3_, 200 mM NaCl) and incubated for 30 minutes on a heat block at 65°C. The cross links were reversed by incubation overnight at 65°C. The eluted material was phenol-extracted and ethanol-precipitated. The DNA was resuspended in 20 μL of water.

#### ChIP-chip analysis

The immunoprecipitated and input DNA was amplified using ligation-mediated PCR [41]. The amplified ChIP DNA was sent to NimbleGen Systems Inc. for labeling and hybridization to a 1.5 kb promoter array containing probes covering the region from -1350 to +150 of 24,275 human genes. (See http://www.nimblegen.com/products/chip/index.html for a description of the promoter array).

The data from the three HNF4α and three AcHis3 promoter array analyses were extracted according to standard operating procedures by NimbleGen Systems Inc. The enrichment of HNF4α IP DNA was calculated as log2 ratio between the HNF4α IP DNA and the genomic input DNA. Mean enrichment ratios for HNF4α at each promoter were calculated by using the five highest values located next to each other in each promoter, as we found that a positive signal typically expanded over five probes. The mean of the three measurements of each promoter and p-values for HNF4α enrichments (Student's t-test; ratios greater than zero) were calculated. Promoters with p-values below 0.05 and at least two-fold enrichments (log2 ratio > 1) of the HNF4α IP DNA were regarded as potential HNF4α target. 1541 promoters had a p-value < 0.05 and a log2 ratio > 1. A list of these genes can be found in additional file 2. Furthermore a searchable Internet database containing the ChIP-chip data from the HNF4α and acetylated histone H3 data is available is available at http://gastro.sund.ku.dk/chipchip/ and at NCBI's GEO http://www.ncbi.nlm.nih.gov/sites/entrez?db=gds under the series accession number: GSE7745.

#### ChIP qPCR analysis

Verification of enrichment due to immunoprecipitation with HNF4α- and HA-antibody was done with real-time PCR using the LightCycler (Roche) according to the manufacturer's protocol with FastStart SYBR Green Master Mix (Roche). Each PCR reaction generated only the expected specific product, as shown by the melting-temperature profiles of final products (dissociation curve, automatically measured by the LightCycler instrument) and by gel electrophoresis of test PCR reactions. The primers listed in additional file 1 were used to amplify human genomic sequences at HNF4α-target loci. Quantification of the ChIP DNA was done using the method described by [42].

### Electrophoretic mobility shift assay (EMSA)

Preparation of nuclear extracts and EMSAs were performed as previously described [39]. For the competition assays, 100-fold (2.5 pmol) excess of unlabeled probes were added to the binding mixtures. 2 μg antibodies against HNF4α sc-8987 (Santa Cruz Biotechnology) were used in the supershift analyses. Oligonucleotides used are listed in additional file 1.

### Bioinformatics

The ChIP-chip data generated with the 1.5 kb promoter array (NimbleGen) and the expression data generated with the Human Genome U133 Plus 2.0 Array (Affymetrix) were coupled by using either accession number or gene symbols provided by the manufacturers. About 2,000 genes that could not be coupled by accession number or gene symbol were linked using the GeneCruiser software http://genecruiser.broad.mit.edu/genecruiser3/. Using these two methods, we were able to link 14,650 NimbleGen promoters from a total of 24,124 promoters to the Affymetrix probesets (ProbeID).

In order to identify transcription factor binding sites over-represented in the promoters that are up-regulated during Caco-2 differentiation, promoter sequences from -1350 to +150 were extracted using the UCSC Table browser http://genome.ucsc.edu/. Over-represented transcription factor binding sites were identified using the PRIMO software http://gastro.sund.ku.dk/primoweb/[28], which is a faster in-house version of the PRIMA software [43]. Promoters enriched in binding to HNF4α were analyzed for transcription factor binding site over-representation using the same procedure (Table 1).

**Table 1:** Analysis for over-representation of transcription factor binding sites using the PRIMO

		HNF4α ChIP-chip data		Caco2 mRNA expression data	
**Acc. No^1^**	**Transcription factor**	**Hits^2^**	**P-value**	**Hits^2^**	**P-value**

M00411	HNF-4alpha1	(3161,18621,452,1089)	1.3E-43	(3296,18493,317,1217)	1.1E-05
M00134	HNF-4	(3294,18488,453,1088)	1.7E-39	(3428,18361,319,1215)	2.2E-04
M00158	COUP-TF, HNF-4	(3343,18439,421,1120)	4.8E-28	(3439,18350,325,1209)	4.1E-05
M00803	E2F	(10649,11133,984,557)	2.0E-27	(10787,11002,846,688)	1.0E-02
M00464	POU3F2	(2673,19109,59,1482)	9.6E-27	(2649,19140,83,1451)	2.4E-15
M00764	HNF-4 direct repeat 1	(3511,18271,418,1123)	5.0E-23	(3613,18176,316,1218)	3.8E-02
M00512	PPARG	(2775,19007,340,1201)	1.5E-19	(2888,18901,227,1307)	1
M00196	Sp1	(8570,13212,777,764)	1.2E-14	(8580,13209,767,767)	2.5E-13
M00108	NRF-2	(2787,18995,321,1220)	1.6E-14	(2937,18852,171,1363)	1
M00932	Sp1	(8850,12932,795,746)	2.7E-14	(8875,12914,770,764)	2.9E-10
M00931	Sp1	(8375,13407,757,784)	1.1E-13	(8377,13412,755,779)	7.6E-14
M00762	PPAR, HNF-4, COUP, RAR	(3936,17846,412,1129)	2.9E-13	(3999,17790,349,1185)	1.5E-02
M00025	Elk-1	(2235,19547,264,1277)	1.4E-12	(2340,19449,159,1375)	1
M00695	ETF	(7391,14391,674,867)	6.9E-12	(7446,14343,619,915)	5.7E-04
M00933	Sp1	(9528,12254,828,713)	1.7E-11	(9544,12245,812,722)	2.1E-09
M00638	HNF-4alpha	(2389,19393,271,1270)	5.5E-11	(2419,19370,241,1293)	7.5E-05
M00032	c-Ets-1(p54)	(2633,19149,291,1250)	8.8E-11	(2730,19059,194,1340)	1
M00763	PPAR direct repeat 1	(3802,17980,387,1154)	1.8E-10	(3860,17929,329,1205)	1.5E-01
M00765	COUP direct repeat 1	(3324,18458,336,1205)	2.9E-08	(3390,18399,270,1264)	1
M00672	TEF	(3512,18270,161,1380)	3.4E-07	(3512,18277,161,1373)	5.0E-07
M00341	GABP	(3601,18181,347,1194)	2.4E-06	(3699,18090,249,1285)	1
M00287	NF-Y	(3353,18429,327,1214)	2.6E-06	(3382,18407,298,1236)	3.8E-02
M00652	Nrf-1	(2721,19061,273,1268)	6.3E-06	(2790,18999,204,1330)	1
M00255	GC box	(9746,12036,803,738)	1.1E-05	(9751,12038,798,736)	1.9E-05
M00430	E2F-1	(2322,19460,236,1305)	3.8E-05	(2369,19420,189,1345)	1
M00224	STAT1	(2188,19594,223,1318)	7.3E-05	(2246,19543,165,1369)	1
M00716	ZF5	(6120,15662,528,1013)	1.8E-04	(6167,15622,481,1053)	1
M00649	MAZ	(9648,12134,782,759)	4.6E-04	(9670,12119,760,774)	4.6E-02
M00309	ACAAT	(3028,18754,286,1255)	5.2E-04	(3056,18733,258,1276)	1
M00775	NF-Y	(2771,19011,265,1276)	5.6E-04	(2793,18996,243,1291)	4.8E-01
M00008	Sp1	(7119,14663,593,948)	1.8E-03	(7071,14718,641,893)	8.1E-11
M00007	Elk-1	(2809,18973,258,1283)	1.4E-02	(2851,18938,216,1318)	1
M00225	STAT3	(2167,19615,206,1335)	1.7E-02	(2205,19584,168,1366)	1
M00778	AhR	(891,20891,98,1443)	3.0E-02	(924,20865,65,1469)	1
M00084	MZF1	(4944,16838,419,1122)	3.4E-02	(4985,16804,378,1156)	1

Analysis for over-representation of transcription factor binding sites using the PRIMO program on the HNF4α target promoters (HNF4α ChIP-chip data), and on the promoters of genes that are up-regulated during intestinal cell differentiation (Caco-2 mRNA expression data).

^1 ^Accession numbers are obtained from Transfac database http://www.biobase.de. ^2 ^The numbers in the parentheses referrers to: The first number = transcription factor binding site (TFBS)-positive promoters in the background set of promoters on the NimbleGen promoter array. The second number = TFBS-negative promoters in the background set of promoters analyzed on the NimbleGen promoter array. The third number = TFBS-positive promoters in the target set of promoters. The fourth number = TFBS-negative promoters in target set of promoters. The background set contains all promoters on the Nimblegen promoter array except the promoters represented in the target set. The target sets of promoters are promoters predicted to associate with HNF4α or promoters up-regulated during Caco-2 differentiation. P-value is calculated using Fisher's Exact test and Bonferroni correction for multiple testing.

The functional terms (GO terms) from the Gene Ontology were associated with promoters on the 1.5 kb promoter array (NimbleGen) through Affymetrix's ProbeIDs and GoSurfer software http://bioinformatics.bioen.uiuc.edu/gosurfer/. GO terms containing a significant over-representation of promoters with two fold HNF4α enrichment in the ChIP-chip analysis were identified using Fishers Exact Test, which was performed using the statistical computing program package R http://cran.r-project.org/. GO terms that were over-represented with a p-value less than 0.1 were included in the analysis. The full list of over-represented GO-terms can be found in additional file 3. A processed list of over-represented GO-terms having positive signals for HNF4α binding in the ChIP-chip analysis was produced (Table 2).

**Table 2:** Distribution of HNF4α targets in selected Gene Ontology categories

***51179 (localization)***
**6810 (transport)** (no)
6869 (lipid transport)(N = 16 E = 9 p = 0.026)
15849 (organic acid transport)(N = 18 E = 10 p = 0.015)
48193 (Golgi vesicle transport)(N = 24 E = 15 p = 0.052)
15837 (amine transport)(N = 16 E = 8 p = 0.012)
6865 (amino acid transport)(N = 15 E = 7 p = 0.014)
***8152 (metabolism)***
**44237 (cellular metabolism) **(no)
6066 (alcohol metabolism)(N = 47 E = 32 p = 0.020)
51186 (cofactor metabolism) (no)
6732 (coenzyme metabolism)(N = 27 E = 19 p = 0.076)
9109 (coenzyme catabolic process)(N = 7 E = 3 p = 0.049)
6084 (acetyl-CoA metabolism)(N = 11 E = 3 p = 0.001)
6091 (generation of precursor metabolites and energy)(N = 92 E = 68 p = 0.007)
6118 (electron transport)(N = 59 E = 41 p = 0.009)
6119 (oxidative phosphorylation)(N = 14 E = 8 p = 0.049)
6082 (organic acid metabolism)(N = 80 E = 54 p = 0.001)
19752 (carboxylic acid metabolism)(N = 80 E = 54 p = 0.001)
9308 (amine metabolism)(N = 55 E = 43 p = 0.073)
6519 (amino acid and derivative metabolism)(N = 47 E = 35 p = 0.057)
6725 (aromatic compound metabolism) (no)
19439 (aromatic compound catabolism)(N = 6 E = 2 p = 0.008)
**43170 (macromolecule metabolism**) (no)
9059 (macromolecule biosynthesis)(N = 124 E = 83 p = 3.5E-05)
6259 (DNA metabolism)(N = 112 E = 80 p = 0.001)
51052 (regulation of DNA metabolism)(N = 10 E = 5 p = 0.039)
6323 (DNA packaging)(N = 46 E = 34 p = 0.054)
6325 (establishment and/or maintenance of chromatin architecture)(N = 46 E = 33 p = 0.034)
6310 (DNA recombination)(N = 18 E = 10 p = 0.027)
6281 (DNA repair)(N = 43 E = 29 p = 0.019)
6260 (DNA replication)(N = 30 E = 22 p = 0.079)
16070 (RNA metabolism)(N = 87 E = 65 p = 0.011)
6399 (tRNA metabolism)(N = 19 E = 10 p = 0.009)
6396 (RNA processing)(N = 69 E = 54 p = 0.051)
**6519 (amino acid and derivative metabolism)(N = 47 E = 35 p = 0.057)**
19538 (protein metabolism) (no)
6412 (protein biosynthesis)(N = 117 E = 75 p = 1.0E-05)
6418 (tRNA aminoacylation for protein translation)(N = 11 E = 5 p = 0.031)
6414 (translational elongation)(N = 7 E = 3 p = 0.034)
6457 (protein folding)(N = 44 E = 31 p = 0.028)
6629 (lipid metabolism)(N = 92 E = 70 p = 0.015)
6631 (fatty acid metabolism)(N = 27 E = 18 p = 0.040)
***50790 (regulation of enzyme activity)***
**43086 (negative regulation of enzyme activity)(N = 14 E = 7 p = 0.025)**
6469 (negative regulation of protein kinase activity)(N = 10 E = 5 p = 0.041)
45859 (regulation of protein kinase activity)(N = 25 E = 17 p = 0.088)
79 (regulation of cyclin dependent protein kinase activity)(N = 12 E = 5 p = 0.011)
6469 (negative regulation of protein kinase activity)(N = 10 E = 5 p = 0.041)

N is the number of identified HNF4α targets assigned with the GO-term indicated. E is the expected number of genes to be found in the GO category. p is the p-value from a Fisher Exact test for the over-representation of HNF4α target genes in the GO category. "no" indicates the higher level GO categories are not over-represented by HNF4α target genes. but contain lower level GO categories with over-representation. A full list of all GO categories with over-representation is presented in additional file 3

## Results

In order to identify transcription factors of importance for the differentiation of the intestinal epithelium, we analyzed the differentiation-dependent expression in Caco-2 cells. The Caco-2 cell line is well established as being one of the best cell lines mimicking the *in vivo *differentiation of enterocytes/colonocytes both morphologically and biochemically [44]. RNA from undifferentiated and differentiated Caco-2 cells were isolated and used to produce hybridization probes for an expression analysis using the Human Genome U133 chip (Affymetrix). This was done to identify the genes that are up-regulated during differentiation. The promoter sequences from genes that were up-regulated more than four times were extracted from the UCSC Genome Bioinformatics Site and used to search for transcription factor binding sites that are statistically over-represented in these promoters. We have recently developed a program called PRIMO that combines the use of transcription factor binding matrices to identify transcription factor binding sites and Fishers Exact Test to analyze for over-representations. The PRIMO analysis demonstrated that up-regulated genes have an over-representation of HNF4α binding sequences or HNF4α-like sequences (COUP-TF, PPAR) and GC-rich sequences (Sp1, E2F, ETF) (Table 1).

**Figure 1:**
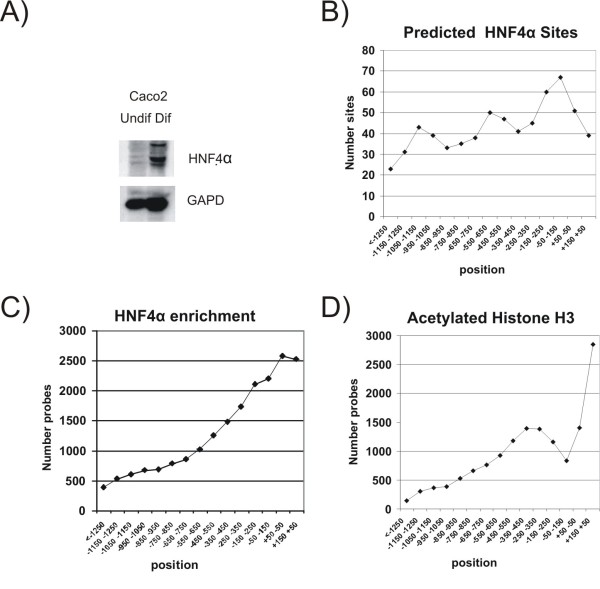
**HNF4α protein level, predicted binding sites and ChIP-chip results for HNF4α and acHis3**. A) Western Blot of HNF4α and glyceraldehyde-3-phosphate dehydrogenase (GAPDH) in nuclear extracts from undifferentiated and differentiated Caco2 cells. B) Distribution of HNF4α binding sites. The positions of HNF4α binding sites were determined in promoters of genes that are up-regulated during differentiation of Caco-2 cells using the M00411 matrix from the Transfac database http://www.biobase.de/ and the PRIMO program http://gastro.sund.ku.dk/primo/. The number of HNF4α hits (Y-axis) was determined in 100 bp intervals from position -1350 to +150 (X-axis). C) Distribution of probes giving a positive signal (> two-fold enrichment) in the HNF4α ChIP-chip analysis. D) Distribution of probes giving a positive signal (>four-fold enrichment) in the acetylated histone H3 ChIP-chip analysis.

An analysis of the level of HNF4α expression during differentiation of Caco2 cells revealed that the HNF4α mRNA level increases 7.8-fold during Caco-2 differentiation in the Affymetrix Gene chip analysis (accessible at http://www.ncbi.nlm.nih.gov/sites/entrez?db=gds; series accession number: GSE7745). Also at the protein level a significant increase in the HNF4α expression was detected (Figure 1A). In order to investigate the role of HNF4α in differentiated intestinal cells, three HNF4α ChIP-chip analyses were performed on differentiated Caco-2 cells. All promoters (from position +150 to -1350) are covered by 15 50'mer oligonucleotide probes on the tiling promoter array. The means of the five highest signals from neighboring probes were used to calculate the mean enrichment of HNF4α IP DNA over genomic DNA for each promoter. Only promoters with significant enrichments (p < 0.05), which were greater than two-fold, were selected in order to limit the set of potential HNF4α target promoters in our further analyses. 1541 promoters contain signals fulfilling these two criteria (see additional file 2). This group contains several promoters that previously have been shown to be regulated by HNF4α in liver (eg. HNF1α, apolipoproteins, etc. [41]). The selected group of potential HNF4α-target promoters have a significant over-representation of HNF4α sites and HNF4α site-like sequences (COUP-TF, PPAR, LEF1) when analyzed by the PRIMO program. GA-rich binding sites (e.g. NRF-2, GABP, Ets) and GC-rich binding sites (e.g. Sp1, E2F, ETF) were also over-represented in the potential HNF4α-target promoters However, we were not able to identify a HNF4α binding site in 2/3 of promoters on the list of potential HNF4α-target promoters, which could be due to either an incomplete identification of the HNF4α binding sites by PRIMO or because HNF4α bound to a region outside the arrayed region has caused isolation of the promoter DNA in the ChIP through protein-protein interactions.

An analysis of the position of the *in silico *identified HNF4α sites revealed that many HNF4α sites are located around the transcriptional start site, but a substantial number of sites are also located further away from the transcriptional start site, particularly around position -500 and -1000 bp (Figure 1B). The analysis of the location of probes giving the ChIP-chip signals from the HNF4α analysis showed the same pattern which is in contrast to the ChIP-chip signals from acetylated histone H3 analysis that are mainly found in the transcribed region (+1 to +150) and the regions around -300 (Figure 1C and 1D).

To identify pathways and functions regulated by HNF4α, we investigated the functional gene ontology (GO) terms that have been assigned to the potential HNF4α-target genes (Table 2). We found that three high-level GO-terms (GO:51179:localization; GO:8152:metabolism; GO:50790:regulation of enzyme activity) contained most of the GO-terms that have an over-representation of HNF4α-target genes. In the group of GO-terms under "localization", we found that genes categorized under the terms lipid transport (GO:6869) and amino acid transport (GO:6865) were over-represented. In the "metabolism" group several GO-terms were over-represented: e.g., amino acid metabolism, lipid metabolism, and DNA and RNA metabolism. In the group "regulation of enzyme activity", "regulation of protein kinase activity" was over-represented. It can be concluded that the HNF4α regulates expression of genes that are essential for the function of the enterocyte: for example, final absorption of nutrients (lipid and amino acid transport and metabolism).

### Intestinal HNF4α-target genes

We selected three promoters for further thorough investigation because they represented different categories of targets for HNF4α regulation.

#### Cdx-2 (CDX2)

**Figure 2:**
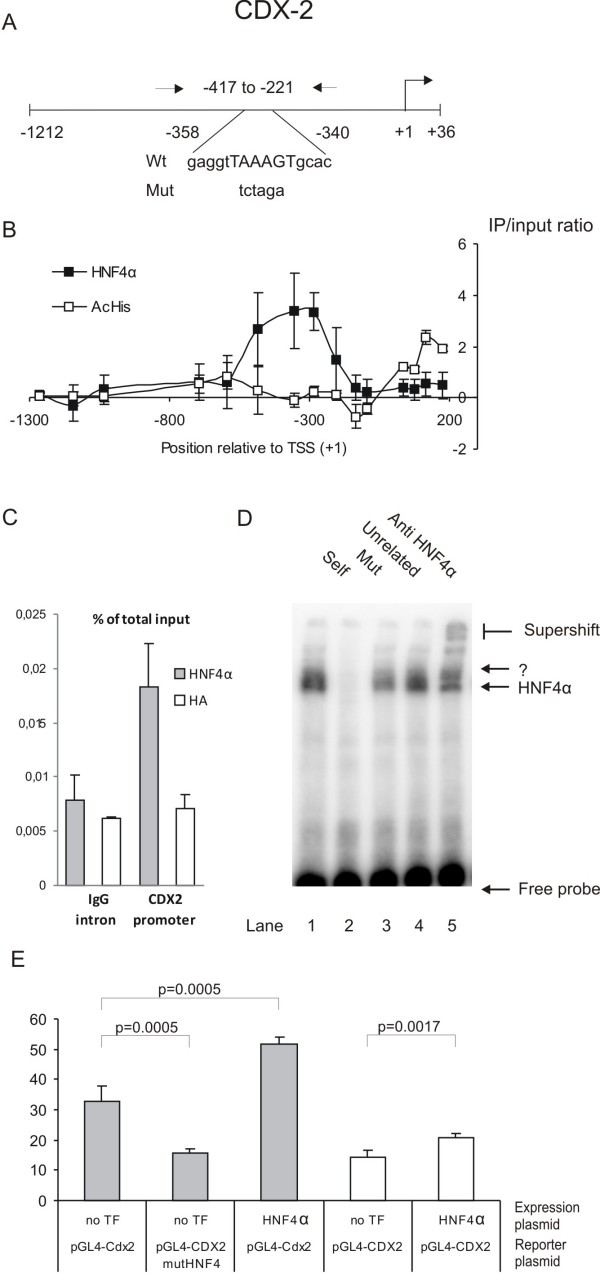
**Analysis of the CDX2 promoter**. A) The promoter region cloned in pGL4.10. The coordinates are relative to +1, the transcriptional start site. The arrows indicate primers used in the real-time qPCR. The possible HNF4α binding site and the mutated sequence is shown below. B) HNF4α ChIP-chip and AcHis3 ChIP-chip results for probes spanning the *CDX2 *promoter. Enrichments are shown as Log2 ratios between immunoprecipitated and input DNA. N = 3. C) Real-time qPCR with *CDX2 *and IgG intron primers using HNF4α ChIP, HA ChIP and input DNA. Enrichments are presented as percent of total input. N = 4. The *CDX2 *promoter enrichment is statistical significant (p-values < 0.05, Student T test). D) Gel shift analysis of the HNF4α site in the *CDX2 *promoter using Caco-2 nuclear extract (lane 1). Competition by unlabelled wt (lane 2), mut-HNF4α oligonucleotides (lane 3) and an unrelated oligonucleotide (lane 4) demonstrating a specific binding. One of the two protein/DNA bands can be supershifted with HNF4α antibody (lane 5). E) Promoter analysis of *CDX2 *promoter in Caco-2 (grey bars) and COS7 (white bars) cells with and without co-transfection of HNF4α expression plasmid. The binding site for HNF4α was mutated in order to analyze the functional importance of the HNF4α site (pGL4-CDX2 mutHNF4). The luciferase activity was corrected for transfection efficiency and normalized to the expression of pGL4-CDX2, *N *= 4.

Interestingly, the ChIP-chip study revealed that the promoter for the intestine-specific transcription factor Cdx-2 is bound by HNF4α. Clear signals from probes located at positions -206, -286, -356, and -486 in the Cdx-2 promoter were retrieved (Figure 2B). The histones on the chromatin of the Cdx-2 gene are furthermore acetylated after the transcriptional start site, indicating that the promoter is transcriptionally active. This data indicates that a transcriptional link exists between HNF4α and Cdx-2. The HNF4α binding to the Cdx-2 promoter was verified by ChIP qPCR analysis using primers covering the HNF4α positive region (Figure 2C). A HNF4α consensus sequence is located at position -358 to -340 (Figure 2A). In order to identify whether the ChIP signals originated from this HNF4α site, we performed a supershift analysis using a probe covering the HNF4α site and nuclear extract from differentiated Caco-2 cells (Figure 2D). Two specific complexes are formed with the probe. One of them is supershifted with an antibody against HNF4α, demonstrating the presence of HNF4α in this complex. To further functionally validate the role of HNF4α in the regulation of Cdx-2 expression, we performed a promoter study of the region -1212 to +36. This region of the promoter was cloned in front of the luciferase reporter gene both as wildtype and with mutations abolishing the HNF4α binding (Figure 2D, lane 3).

The Cdx-2 promoter constructs were used to transfect the intestinal epithelial cell line Caco-2 and COS7 cells, a cell line without detectable HNF4α expression and previously used to study the effect of HNF4α on promoter activity [45,46]. The Cdx-2 promoter was able to drive a higher reporter gene expression in Caco-2 compared to COS7 cells. Over-expression of HNF4α significantly increased the reporter gene expression in both Caco-2 and COS7 cells, and mutation of the HNF4α site significantly reduced the reporter gene expression in Caco-2 cells to the basic level observed in COS7 cells (Figure 2E).

#### Trehalase (TREH)

The trehalase promoter is another interesting promoter that is on the list of potential HNF4α-target genes (see additional file 2). Trehalase is a brush border disaccharidase that hydrolyses trehalose, mainly found in fungi, to glucose [47]. Trehalase has a very restricted expression pattern, as it is only expressed in the mature enterocytes in the small intestine after weaning and in the kidney [48]. Trehalase is therefore regarded as a specific marker for the terminally differentiated enterocyte.

**Figure 3:**
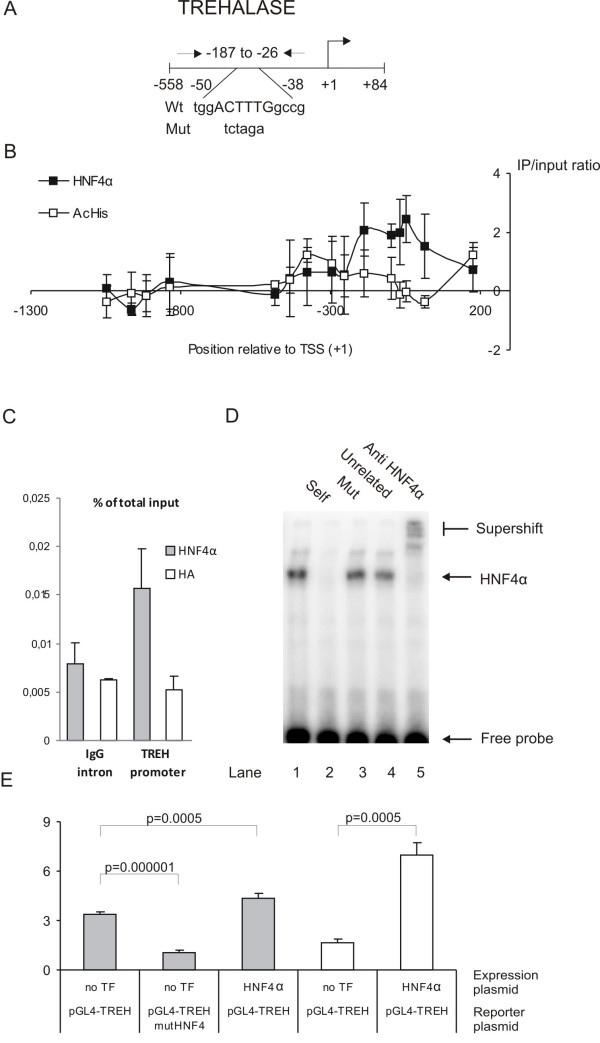
**Analysis of the trehalase (*TREH*) promoter**. A) Map of the *TREH *promoter region. PCR primers, the HNF4α binding site, and the mutations are shown. B) HNF4α ChIP-chip and AcHis3 ChIP-chip results for probes spanning the *TREH *promoter. N = 3. C) Real-time qPCR analysis of HNF4α ChIP, HA ChIP and input DNA using *TREH *and IgG intron primers. N = 3. The *TREH *promoter enrichment is statistical significant (p-values < 0.05, Student T test). D) Gel shift analysis of the HNF4α site in the *TREH *promoter. E) Promoter analysis of the *TREH *promoter in Caco-2 (grey bars) and COS7 (white bars) cells with and without co-transfection of HNF4α expression plasmid and a mutation in the HNF4α site (pGL4-TREH mutHNF4). The luciferase activity was corrected for transfection efficiency and normalized to the expression of pGL4-TREH, *N *= 4.

An HNF4α ChIP-enrichment was detected by the probes in positions +14, -46, -66, -96, and -186 (Figure 3B). In this region we also found a significant histone acetylation. A predicted HNF4α binding site is located at position -50 to -38, and a ChIP qPCR analysis of the region confirmed the HNF4α interaction with this region (Figure 3C). A supershift analysis clearly demonstrated a strong specific HNF4α binding to the predicted HNF4α site (Figure 3D), and a functional reporter gene analysis of the trehalase promoter in Caco-2 and COS7 cells showed that the trehalase promoter is strongly influenced by both HNF4α over-expression and by mutations in the HNF4α binding site (Figure 3E). The results point toward a strong involvement of the HNF4α in the regulation of trehalase expression.

#### Cingulin (CGN)

**Figure 4:**
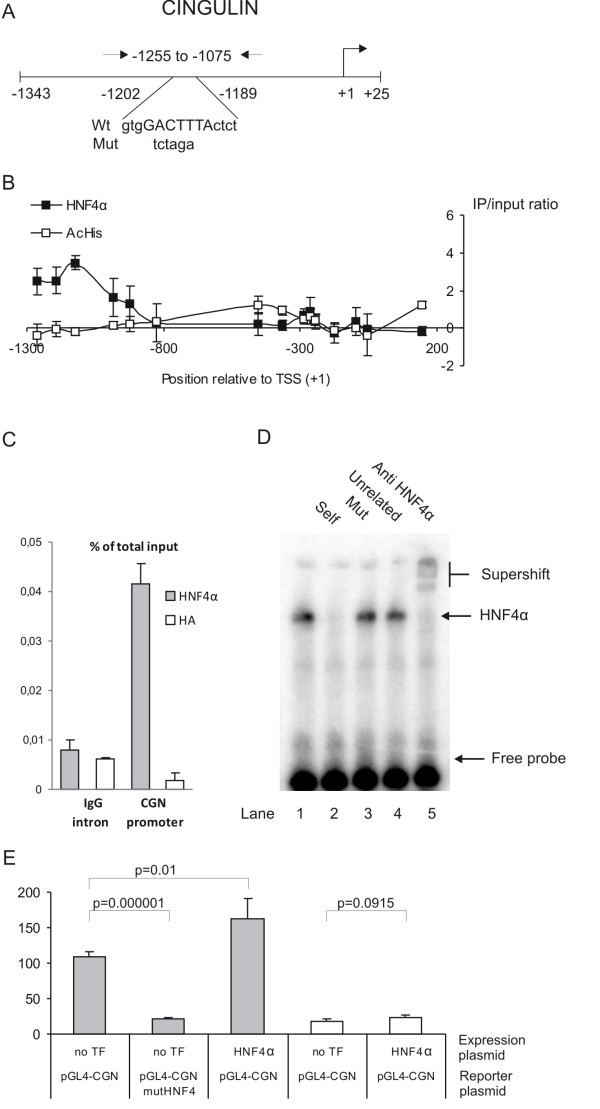
**Analysis of the cingulin (*CGN*) promoter**. A) Map of the *CGN *promoter region. PCR primers, the HNF4α binding site, and the mutations are shown. B) HNF4α ChIP-chip and AcHis3 ChIP-chip results for probes spanning the CGN promoter. N = 3. C) Real-time qPCR analysis of HNF4α ChIP, HA ChIP and input DNA using *CGN *and IgG intron primers. N = 3. The *CGN *promoter enrichment is statistical significant (p-values < 0.05, Student T test). D) Gel shift analysis of the HNF4α site in the *CGN *promoter. E) Promoter analysis of the *CGN *promoter in Caco-2 (grey bars) and COS7 (white bars) cells with and without co-transfection of HNF4α expression plasmid and a mutation in the HNF4α site (pGL4-CGN mutHNF4). The luciferase activity was corrected for transfection efficiency and normalized to the expression of pGL4-CGN, *N *= 4.

The function of cingulin is presently not fully understood, but the cingulin protein has been reported to be present in tight junctions interacting with the tight junction protein ZO-1 [49]. The involvement of cingulin in tight junction formation prompted us to further investigate the regulatory role of HNF4α in cingulin expression, as tight junction formation is an important event in the differentiation of the enterocyte and the establishment of the barrier between the intestine and the intestinal lumen. The ChIP-chip analysis revealed a binding of HNF4α to the region from -1264 to -924 in the cingulin promoter (Figure 4B). This binding was confirmed by ChIP qPCR (Figure 4C). We also found that the chromatin of the cingulin promoter is histone H3-acetylated. However, the acHis3 signal does not directly overlap the HNF4α signal (Figure 4B).

A potential HNF4α binding site is located at position -1202 to -1189 (Figure 4A). EMSA demonstrated that this site specifically forms a protein/DNA complex that is supershifted by a HNF4α antibody (Figure 4D). Mutations in the HNF4α site reduced the promoter activity in Caco-2 cells, and over-expression of HNF4α increased the promoter activity in Caco-2 cells, however in COS7 cells the reporter gene expression is only slightly influenced by HNF4α over-expression (Figure 4E). The over-expression results demonstrate that HNF4α plays a role in regulating cingulin expression, but other transcription factors are important for the activation of the cingulin gene, which are not expressed in COS7 cells. The mutation of the HNF4α site reduces the cingulin promoter activity in Caco-2 cells to the basic level observed in COS7 cells.

### In vivo binding of HNF4α in mouse small intestine

**Figure 5:**
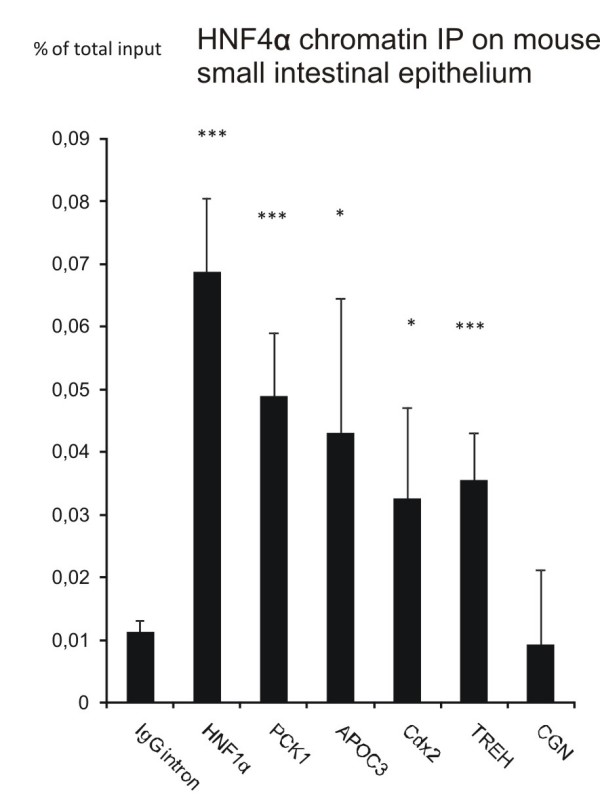
**HNF4α ChIP on mouse small intestinal epithelium**. Chromatin immunoprecipitation analysis of HNF4α binding to HNF1α (*Tcf1*), phosphoenolpyruvate carboxykinase 1 (*Pck1*), apolipoprotein C3 (*Apoc3*), Cdx-2 (*Cdx2*), trehalase (*Treh*), and cingulin (*Cgn*) promoters in mouse small intestinal epithelium. An intron in the IgG gene served as a negative control (IgG). The analyzed promoter regions are all conserved between human and mouse. Enrichments are represented as percent of the total amount of genomic input DNA in ChIP. Significant enrichments are indicated (p-values < 0.001 is shown by ***, p-values < 0.05 is shown by *).

In order to further verify the HNF4α binding to the Cdx-2, trehalase, and cingulin promoters *in vivo*, ChIP analyses were performed on mouse small intestinal mucosa (figure 5). As positive controls HNF4α (Tcf1), apolipoprotein CIII (Apoc3), and phosphoenolpyruvate carboxykinase 1 (Pck1) were also included in the analysis as these genes have been reported to be HNF4α targets in the mouse liver[50] and were on our list of potential HNF4α targets in Caco-2 cells (see additional file 2). HNF1α, Apoc3, and Pck1 promoters were all significantly enriched for HNF4α IP DNA, as well as Cdx2 and trehalase. However, the HNF4α ChIP analysis of the cingulin promoter did not reveal a binding of HNF4α in the mouse small intestine.

## Discussion

The HNF-transcription factors are recognized as being central factors in the regulation of liver- and pancreas-specific genes. Odom et al. (2004) performed ChIP-chip analysis of HNF1α, HNF4α, and HNF6 in hepatocytes and pancreatic islets and found that HNF4α was bound to 12% of the genes in hepatocytes and 11% in pancreatic islets. We have found that 11% of the genes that are up-regulated in the gene expression array analysis during differentiation have a two-fold enrichment of HNF4α IP DNA in the ChIP-chip of the Caco-2 cells and the bioinformatic analyses showed that HNF4α binding sites are significantly over-represented in promoters that are up-regulated during Caco-2 differentiation (table 1). We have previously analyzed intestinal differentiation in mice and identified HNF4α as a regulator of differentiation-induced transcription [28]. These results support the suggestion that HNF4α is a central player in the differentiation-induced expression of genes in the gastrointestinal tract [28,30,41].

By assigning biological functions (GO-terms) to the HNF4α target genes, we have identified that HNF4α target genes are over-represented in the GO-terms related to lipid and amino acid transport and metabolism. Absorption of nutrients is one of the main functions of the small intestinal epithelium. It is notable that HNF4α binds to many apolipoprotein promoters (see the ChIP-chip database: http://gastro.sund.ku.dk/chipchip/; search term = apoliprotein). The HNF4α regulation of many genes involved in metabolism agrees with the notion that the transcriptional activity of HNF4α is regulated by AMP-activated protein kinase, the central component of a cellular signaling system that regulates many metabolic enzymes and pathways in response to reduced intracellular energy levels [34].

Another important role of the enterocytes is the final hydrolysis of oligopeptides to dipeptides and amino acids by the membrane-bound brush border peptidases. Our ChIP-chip analysis shows that several of the important brush border peptidases can be expected to be regulated by HNF4α: e.g., aminopeptidase N (*ANPEP*), which is one of the most abundant proteins in the brush border. A 4.3- fold enrichment of HNF4α IP DNA on aminopeptidase N promoter was found and the aminopeptidase N mRNA expression is up-regulated 32-times during Caco-2 differentiation (see additional file 2). We have previously studied the aminopeptidase N promoter in detail[16,28,51-53] and have identified a COUP/HNF4α binding site, which is important for epithelial expression. We originally suggested that the site interacts with the COUP transcription factor [52]. However, as COUP and HNF4α have overlapping binding specificities, we find it likely that HNF4α binds to the COUP/HNF4α element.

Expression of disaccharidases in the brush border membrane is a unique characteristic of the enterocytes. Disaccharidase expression is not found in the liver or in the pancreas. The expression of sucrase-isomaltase (*SI*), lactase-phlorizin hydrolase (*LCT*), trehalase (*TREH*), and maltase-glucoamylase (*MGAM*) are necessary for digesting sucrose, lactase, trehalose, and maltose, respectively, and deficiency in the expression of any of these enzymes results in severe digestive problems [54,55]. We have previously described that the lactase-phlorizin hydrolase promoter activity is regulated by HNF4α through binding to an enhancer located 13910 bp from the promoter [56]. This region was not analyzed in ChIP-chip, but we found a significant binding to the trehalase promoter in ChIP-chip analysis (Figure 3B), which was verified by ChIP-qPCR in both Caco-2 cells (Figure 3C) and mouse small intestinal mucosa (Figure 5). EMSA and promoter analyses of the trehalase promoter further demonstrate that HNF4α binds and regulates the trehalase promoter activity (Figure 3DE). These results show that HNF4α is involved in regulating dissacharidase expression specific to the small intestinal epithelium.

The differentiation of the absorptive intestinal cell (enterocyte/Caco-2) is accomplished by a remarkable phenotypic change. During differentiation the enterocyte becomes polarized with tight junctions and a brush border membrane with a highly specialized content of digestive enzymes. HNF4α seems to directly regulate some of the genes involved in this process. For example, cingulin is a tight junction associated protein that we find is regulated by HNF4α in Caco-2 cells [49]. We have not, however, been able to demonstrate HNF4α binding to the mouse cingulin promoter in the small intestinal mucosa, which indicates that either HNF4α is not regulating cingulin in the mouse or that an HNF4α binding site in the mouse cingulin promoter is located outside the investigated region. Interestingly, it has recently been found that more genes are regulated by HNF4α in human liver than in mouse [50]. Only 1/3 of the HNF4α bound human promoter genes were also found in mouse. Thus, based on our EMSA and ChIP results on the Caco-2 cells we suggested that the cingulin is a *bona fide *HNF4α target gene in Caco-2 cells. Other tight junction proteins, claudins (*CLDN2*[27]), are on the list of potential HNF4α-target genes. The findings of HNF4α controlling the expression of genes involved in morphological differentiation become even more important when interpreted with the finding by Peignon et al where E-cadherin is shown to control the nuclear abundance of HNF4α [57]. This could place HNF4α as an important link between the changes in morphology and gene expression during differentiation of intestinal cells.

As HNF4α could have a specific role in intestinal expression which is different from its role in the hepatocytic and pancreatic expression, we sought for HNF4α target genes that are specifically involved in intestinal development. A search in the ChIP-chip data for transcription factors that could be linked to HNF4α regulation revealed that HNF4α binds to the Cdx-2 promoter. Cdx-2 plays a fundamental role in the mammalian embryonic development. It controls the specification of the trophectoderm lineage, the first differentiation event after fertilization of the egg [58], and the anteroposterior patterning and posterior axis elongation [59]. However, in adult mammals the Cdx-2 expression is restricted to the small and large intestine [36,60]. Our functional analyses show that HNF4α regulates Cdx-2 promoter activity in Caco-2 (figure 2). This is supported by the enrichment of HNF4α IP DNA to the Cdx-2 promoter in the ChIP qPCR analysis of the mouse small intestinal mucosa (figure 5). In support of our finding Benahmed et al. (2008) have shown that HNF4α is involved in the regulation of the mouse Cdx-2 gene transcription [61]. In order to further validate the importance of HNF4α for the Cdx-2 promoter activity, we have, however unsuccessfully, tried to inhibit the HNF4α activity by RNAi (transfection with HNF4α shRNA plasmids and siRNA) and by overexpression of a dominant negative version of HNF4α [62] in Caco-2 cells (personal data). The reduced HNF4α level seems to cause a poor attachment of the cells to the culture disc suggesting that alterations of the cell-cell or cell-substratum interactions have occurred. Similar problems have previously been reported when Caco-2 cells with reduced Cdx-2 expression were produced [63]. Ablation of the HNF4α gene in fetal mouse colon resulted in failure to produce normal crypts and influencing the expression of several intestinally expressed genes [27]. A comparison of these genes with our ChIP-chip data revealed HNF4α ChIP signals in the promoters of SULT1B and MUCDHL genes, thus verifying the results by Garrison *et al*., 2008. However, other reported HNF4α target promoters (ALDOB, APOC2, MUC3, SLC39A[27]) were unfortunately not represented by probes in the promoter regions on the Nimblegen promoter array and these promoters were therefore not included in the ChIP-chip analysis.

Several intestinal expressed genes are regulated by a combination of Cdx-2 and HNF1α: e.g., sucrase-isomaltase [4,13] and lactase-phlorizin hydrolase [3,64]. It is well known that a transcriptional circuit exists between HNF1α and HNF4α in liver, as the HNF1α promoter is a target for HNF4α regulation and HNF1α regulates the HNF4α expression [41].

## Conclusion

**Figure 6:**
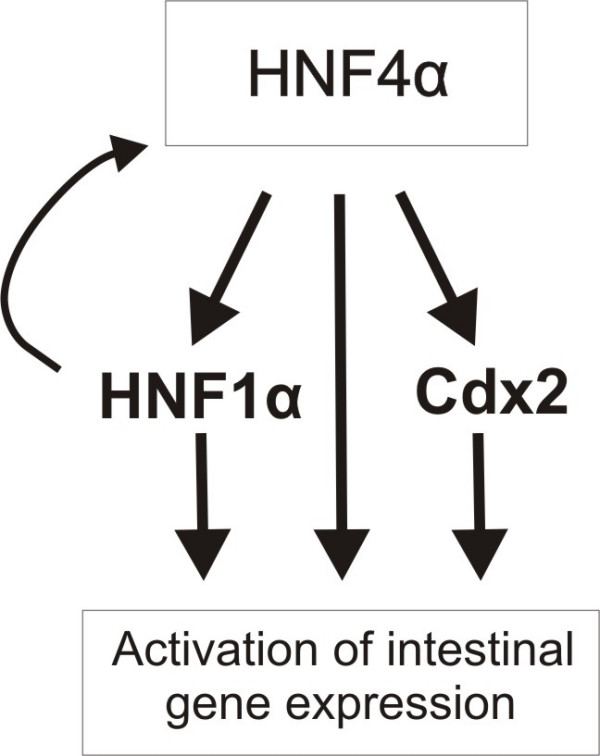
**A model of some of the components in an HNF4α regulated transcription factor network regulating intestinal expression of genes during cellular differentiation**.

The HNF4α ChIP-chip analysis of differentiated Caco-2 cells has substantiated the role of HNF4α as a key transcription factor in the differentiation-dependent regulation in intestinal epithelial cells. Based on our results (table 3), we suggest that HNF4α is a transcriptional node that is involved in the maintenance of the network of transcription factors (Cdx-2 and HNF1α) in intestinal epithelial cells (Figure 6) although additional factors are necessary to specify the intestinal specific expression. In combination with the other members of the network, HNF4α regulates expression of the genes that give the intestinal cell its unique biochemical and physiological features: e.g., trehalase and cingulin. In future studies it would be very interesting to examine the role of HNF4α in the initiation of the differentiation process

**Table 3:** Summary of the evidence for HNF4α regulation of intestinally expressed genes

Gene (*Gene symbol*)	mRNA up-regulation during^1^	ChIP-chip^2^	ChIP q-PCR^3^	ChIP q-PCR^3^
	Caco-2 differentiation	Caco2	Caco2	Mouse
Cdx-2 (*CDX2*)	3.0	5.0	1.9	2.9
Trehalase (*TREH*)	3.5	4.1	1.9	3.2
Cingulin (*CGN*)	3.1	4.9	4.3	0.3
Apolipoprotein CIII (*APOC3*)	9.9	5.3	NA	3.9
HNF1α (*TCF1*)	1.6	4.7	NA	6.2
Phosphoenolpyruvate	7.7	5.6	NA	4.4
carboxykinase 1 (*PCK1*)				

^1^Fold up-regulation of mRNA during Caco2 cell differentiation. Data were obtained from gene array expression analysis of undifferentiated and differentiated Caco2 cells.^2^ChIP-chip enrichment of HNF4α target DNA compared to genomic DNA. The data are presented as fold enrichments between HNF4α immunoprecipitated DNA and genomic DNA. ^3^q-PCR analysis of the HNF4α immunoprecipitated DNA shown as fold enrichment of the HNF4α IP DNA compared to genomic DNA (IgG intron). Array data can be downloaded from NCBI's GEO http://www.ncbi.nlm.nih.gov/sites/entrez?db=gds under the series accession number: GSE7745.

## Competing interests

The authors declare that they have no competing interests.

## Authors' contributions

MB carried out the HNF4α ChIP, transfections, EMSAs, qPCR, and helped to draft the manuscript. SB designed ChIP protocol. JM amplified the HNF4α ChIP DNA for ChIP-chip analysis and performed qPCR. JO performed the gene expression array analysis on the Caco2 cells and provided his expertise in drafting the manuscript. JTT conceived the idea of the study, coordinated the study, made the bioinformatic analysis of the array data and drafted the manuscript. All authors read and approved the final manuscript.

## Pre-publication history

The pre-publication history for this paper can be accessed here:

http://www.biomedcentral.com/1471-230X/9/68/prepub

## Supplementary Material

Additional file 1 **Oligonucleotide lists**. The complete lists of oligonucleotides used in the experiments. Click here for file

Additional file 2 **The list of potential HNF4α target genes in differentiated Caco2 cells**. See first sheet. Click here for file

Additional file 3 **Over-represented GO-terms**. Full list of over-represented GO-terms. Click here for file
