# Supplementary material for: Mapping of HNF4α target genes in intestinal epithelial cells
Source: BMC Gastroenterol. 2009 Sep 17;9:68. doi: 10.1186/1471-230X-9-68 (PMC2761415; doi:10.1186/1471-230X-9-68)
Supplement: Additional file 1 — Oligonucleotide lists. The complete lists of oligonucleotides used in the experiments. [file 1471-230X-9-68-S1.DOC]

Additional file 1

Primers used in ChIP qPCR

| Refseq (Gene) | Sense primer (5’-3’ direction) | Antisense primer (5’-3’ direction) |
| --- | --- | --- |
| NM_001265 (CDX2) | gccttttctcttcccctctc | cccctcgaaacataatttgc |
| NM_007180 (TREH) | gcaccagctgagactgtgaa | cacagcctggctcacctg |
| NM_020770 (CGN) | tacagcttgctctggaatgc | cctgtatctattggagtagaagaggaa |
| Mouse primers | Sense primer (5’-3’ direction) | Antisense primer (5’-3’ direction) |
| CDX2 | aggcgtttgcaagtctcttc | tacgagcttcctccttccaa |
| TREH | caggtggccacacctcatag | acctctggcttgctgaacac |
| CGN | ttcacaaaaacgtgcaatgt | cgcctagtcagccgagtatag |
| TCF1 (HNF1α) | gcaaggctgaagtccaaagt | attggagctggggaaattct |
| APOCIII | gtgaaaagcatgggcaatct | agtccagaccagagcctgag |
| PCK1 | taaggcaagagcctgcagtt | taaggcaagagcctgcagtt |

Primers used in promoter cloning and site-directed mutagenesis.

| Refseq (Gene) | Sense primer (5’-3’ direction) | Antisense primer (5’-3’ direction) | Sense mutational primer (5’-3’ direction) | Antisense mutational primer (5’-3’ direction) |
| --- | --- | --- | --- | --- |
| NM_001265 (CDX2) | gctcgagCAGCGCTTCCCAAACCAAGA | caagcttGGCTCCTCGCGGCTCTTCT | CTGTGATTGGAGGTtctagaGCACCAGGTTGGAAGGAGGAAG | CTTCCAACCTGGTGCtctagaACCTCCAATCACAGGTTCAAAG |
| NM_007180 (TREH) | ctcgagATTACAGGTACCCGCCATCAT | aagcttCAGCCCCAGCAGCAGTAG | CGCCTCAAAGGCTGGtctagaGCCGACTTGGACAGGCAGGTGA | CCTGTCCAAGTCGGCtctagaCCAGCCTTTGAGGCGTGGCCACC |
|  |  |  |  |  |
| NM_020770 (CGN) | ctcgagCATTTCCTCTTCTGTCCCCACCCCCACTAC | agatctAGCTCCGGCCCTCCCCCTCGTCCTC | GGAGCTGGTAAGTGtctagaACTCTGTAGGACCCACACCTAGC | GTGGGTCCTACAGAGTtctagaCACTTACCAGCTCCTCAGCTG |

Oligos used in EMSA

| Refseq (Gene) | Wild type oligos 5’-3’  (position covered relative to +1) | | Mutated oligos 5’-3’  (position covered relative to +1) | |
| --- | --- | --- | --- | --- |
| NM_001265 (CDX2) | gtgattggaggttaaagtgcaccaggttg | ccaacctggtgcactttaacctccaatca | CTGTGATTGGAGGTtctagaGCACCAGGTTGGAAGGAGGAAG | CTTCCAACCTGGTGCtctagaACCTCCAATCACAGGTTCAAAG |
|  | (-363 to -334) | | (-373 to -324) | |
| NM_007180 (TREH) | cctcaaaggctggactttggccgacttgg | tccaagtcggccaaagtccagcctttgag | CGCCTCAAAGGCTGGtctagaGCCGACTTGGACAGGCAGGTGA | CCTGTCCAAGTCGGCtctagaCCAGCCTTTGAGGCGTGGCCACC |
|  | (-140 to -111) | | (-150 to -101) | |
| NM_020770 (CGN) | gctggtaagtggactttactctgtaggac | ggtcctacagagtaaagtccacttaccag | GGAGCTGGTAAGTGtctagaACTCTGTAGGACCCACACCTAGC | GTGGGTCCTACAGAGTtctagaCACTTACCAGCTCCTCAGCTG |
